# Simultaneous Analysis of Twelve Bile Acids by UPLC-MS and Exploration of the Processing Mechanism of Bile Arisaema by Fermentation

**DOI:** 10.1155/2019/2980596

**Published:** 2019-09-09

**Authors:** Qimiao Zhao, Guoshun Shan, Dan Xu, Hui Gao, Ji Shi, Chengguo Ju, Guimei Lin, Fan Zhang, Tianzhu Jia

**Affiliations:** School of Pharmacy, Liaoning University of Traditional Chinese Medicine, Dalian, China

## Abstract

Ultrahigh-performance liquid chromatography (UPLC) coupled with quadrupole time-of-flight tandem mass spectrometry (Q/TOF-MS) in the MS/MS mode and UPLC coupled with triple quadrupole mass spectrometry (QqQ-MS) using the multiple reaction monitoring (MRM) mode were used to make a qualitative and quantitative analysis of twelve bile acids in Bile Arisaema. The fragmentation pathway of twelve bile acids was proposed. The quantification method showed a good linearity over a wide concentration range (*R*^2^ > 0.99), repeatability (RSD < 4.12%), stability (RSD < 4.25%), precision (RSD < 4.06%), and recovery (95.36–102.15%). Content of twelve compounds in Bile Arisaema varied significantly depending on region. Chemometric methods, hierarchical clustering analysis (HCA), and principal components analysis (PCA) were successfully used to optimize the fermentation time of the Bile Arisaema. The results suggested that the Bile Arisaema could complete fermentation in 15 days. The possible processing mechanism of Bile Arisaema promoted the transformation of conjugated bile acids into free bile acids in fermentation.

## 1. Introduction

Fermentation is one of the traditional processing technologies commonly used in Traditional Chinese Medicine (TCM) for enhancing efficacy, producing new bioactivities, and alleviating toxicity [1, 2]. Bile Arisaema (BA), the fermented product of the Rhizoma Arisaematis with pig bile, has been traditionally used for clearing heat and reducing phlegm in TCM theory for more than one thousand years (since the Song Dynasty) [3, 4]. In addition, it is widely used as folk medicine in Korea for treating rheumatism, ulcer of the digestive tract, and cancer. Pharmacology has demonstrated the anti-inflammatory effect of BA in lipopolysaccharide inducing phorbol 12-myristate 13-acetate-differentiated THP-1 macrophages [5]. Besides, increasing research studies have demonstrated that BA has the analgesic and sedative effects. In fact, more than 25 kinds of Chinese patent medicine preparations using BA as the main raw material are recorded in Chinese Pharmacopoeia (2015), including well-known Xiaohuoluo pills, Xiaoer Zhibao pills, and Xiaoer Zhisou syrup [3]. However, so far, there is no rapid and precise content determination method in Chinese Pharmacopoeia (2015) to control the quality of BA [3]. It is still mainly based on human experience evaluation, which is easily influenced by subjective and external environmental factors lacking objectivity and authenticity. Because of the high economic benefit and deficiency of quality standard, the adulteration problem of BA is alarming in Chinese markets. Therefore, it is necessary to establish rational quality control methods of the BA.

At present, bile acids derived from the pig bile are considered as the main active components in the BA [6]. In our previous study, the discrepancy was found in the content of bile acids of bile in different animals and the efficacy of clearing heat and reducing phlegm was also different [7, 8]. The result also reminds that the content of bile acids was related to the efficacy of clearing heat and reducing phlegm in BA. In fact, thin layer chromatography (TLC) [9] and high-performance liquid chromatography (HPLC) [10, 11] had been performed to conduct qualitative and quantitative analysis of bile acids in BA. However, due to the low content and weak UV absorbance of the bile acids, these routine detective methods were not sensitive and selective enough to determine the minor or trace bile acids in BA [10]. Fortunately, due to its high resolution, sensitivity, and accuracy, UPLC-Q/TOF-MS/MS has become a dominant tool to analyze the chemical components of TCM. It can also provide isotopic abundances and the elemental composition of fragment ions which are greatly valuable to the structural analysis of ingredients [12, 13]. Furthermore, ultraperformance liquid chromatography coupled with tandem mass spectrometry (UPLC-QqQ-MS/MS) in the multiple-reaction monitoring mode (MRM) has been developed as a convenient and time-saving method for quantitative analysis of various compounds because of the remarkable separation effect of UPLC and the high sensitivity of tandem mass spectrometry [14–18]. Therefore, UPLC-Q/TOF-MS/MS and UPLC-QqQ-MS/MS are suitable for qualitative and quantitative analysis of bile acids in Bile Arisaema.

In addition, standard processing technology and explicit processing principle are the key to guarantee the clinical efficacy of TCM. However, there are rare researches on the processing technology and mechanism in the BA [19]. It is even only vague description technology parameters of BA in Chinese Pharmacopoeia (2015) [3]. Therefore, it is necessary to optimize the processing technology and explore the processing mechanism of BA. But, the minor differences between very similar chromatograms might be missed due to complex multivariate data sets for the complicated composition of TCM. It also makes a challenge to explore the processing mechanism. However, chemometric approaches have been increasingly viewed as valuable complements to UPLC-MS/MS practices because a large number of variables can be simultaneously controlled to achieve the expected separations [20, 21]. Accordingly, the combination of UPLC-MS/MS analysis and chemometrics would be a powerful tool to optimize the fermentation time and explain the processing principle of BA.

In this work, UPLC-Q/TOF-MS/MS was employed to confirm the bile acids in the methanolic extract of Bile Arisaema. The fragmentation behavior of bile acids was also explored in the negative mode. Then, an UPLC-QqQ-MS/MS method in the MRM mode was established to determine the content of twelve active components in different origins and fermentation times (0 day to 30 days) of BA. It could be used to evaluate the quality and explore the processing mechanism of BA. This study will serve as the first example of comprehensive quality assessment and processing mechanism analysis in Bile Arisaema.

## 2. Experimental

### 2.1. Materials and Reagents

LC-MS grade formic acid was supplied from Merck KGaA (Darmstadt, DE). Methanol and acetonitrile were supplied from Fisher Scientific (Watham, MA, USA). Deionized water was obtained by a Mill-Q system (Billerica, MA, USA). Other chemicals were of analytical purity.

**Figure 1:**
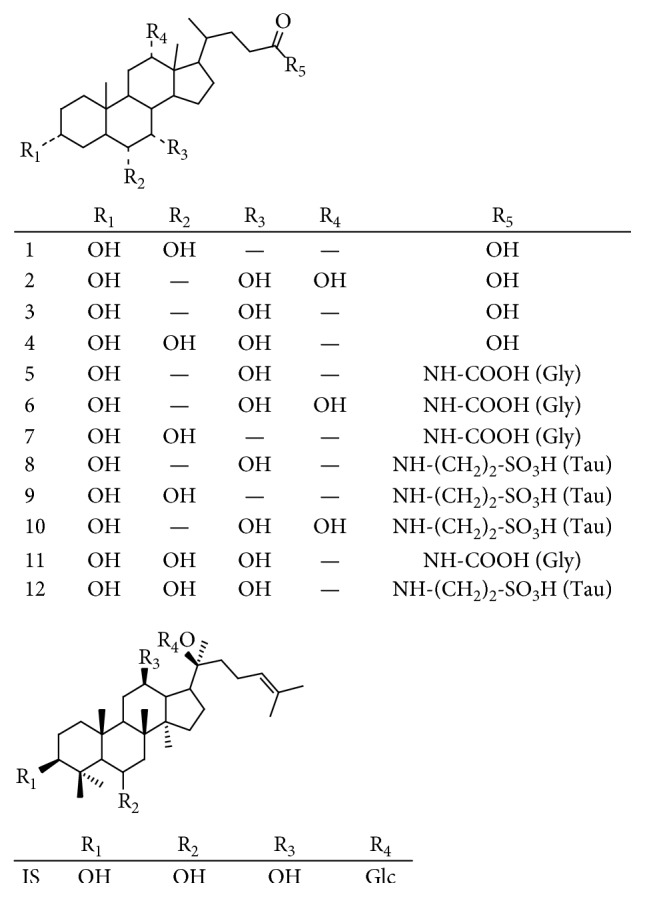
The chemical structures of twelve bile acids and IS (internal standard).

Reference standards including hyodeoxycholic acid (HDCA), cholic acid (CA), chenodeoxycholic acid (CDCA), hyocholic acid (HCA), glycochenodeoxycholic acid (GCDCA), glycocholic acid (GCA), glycohyodeoxycholic acid (GHDCA), taurochenodeoxycholic acid (TCDCA), taurohyodeoxycholic acid (THDCA), taurocholic acid (TCA), glycohyocholic acid (GHCA), and taurohyocholic acid (THCA) were isolated by our library. Their structures were elucidated on the basis of the results of NMR, MS, and IR spectroscopic analysis and compared with the precious references [22, 23]. The purity of these reference standards was all above 98%. Their structural information is shown in Figure 1.

The roots and rhizomes of *Arisaema amurense* Maxim (Rhizoma Arisaematis) were collected from Benxi City (41°40′–41°50′N; 123°46′–123°32′E), Liaoning Province, China, in November 2017 and authenticated by Professor Yan-Jun Zhai of Liaoning University of Traditional Chinese Medicine. The voucher specimen (no. 20171105) was deposited in the Key Laboratory of Processing, School of pharmacy, Liaoning University of Traditional Chinese Medicine, Dalian, China.

The bile of pigs was purchased from Dalian Chu-Ming meat federation Co., Ltd., Dalian, Liaoning, China. The twenty batches of Bile Arisaema were collected in December of 2017 from Sichuan, Hebei, Beijing, Anhui provinces of China. The samples were also authenticated by Professor Yan-Jun Zhai. The voucher specimens (no. 20171201–201711220) were deposited in the Key Laboratory of Processing, School of pharmacy, Liaoning University of Traditional Chinese Medicine, Dalian, China. Details of the samples are listed in Table 1.

### 2.2. Preparation of Standard Solutions

Appropriate amounts of HDCA, CA, CDCA, HCA, GCDCA, GCA, GHDCA, TCDCA, THDCA, TCA, GHCA, and THCA were separately weighed and dissolved in methanol to get the stock solutions. Then, the twelve stock solutions were mixed and diluted with methanol to prepare a final mixed standard solution containing 8.8 *μ*g/mL of HDCA, 6.5 *μ*g/mL of CA, 16.9 *μ*g/mL of CDCA, 7.8 *μ*g/mL of HCA, 19.7 *μ*g/mL of GCDCA, 6.0 *μ*g/mL of GCA, 6.3 *μ*g/mL of GHDCA, 21.9 *μ*g/mL of TCDCA, 27.2 *μ*g/mL of THDCA, 10.6 *μ*g/mL of TCA, 5.6 *μ*g/mL of GHCA, and 8.9 *μ*g/mL of THCA, respectively. A series of working solutions of these ingredients were obtained by diluting mixed standard solution with methanol at the appropriate concentrations. All the solutions were filtered through a 0.22 *μ*m filter membrane prior to injection and stored at 4°C.

### 2.3. Sample Preparation

The samples were grinded into powder less than 100 meshes by a pulverizing machine. About 2.0 g of sample powder was weighed accurately into a 100 mL conical flask with cover, and 50 mL methanol was added. After accurate weighing, the mixture was sonicated (power, 250 W; frequency, 50 kHz) for 30 min (Kunshan ultrasonic equipment Co., Ltd, Jiangsu, China). The extracted solution was cooled to room temperature and made up to the original weight with methanol. The supernatants were filtered through a 0.22 *μ*m filter membrane prior to injection and stored at 4°C.

### 2.4. UPLC-Q/TOF-MS/MS Conditions

The UPLC-Q/TOF-MS/MS analysis was carried out on an Acquity I-Class UPLC system (Waters Corp., Milford, MA, USA) coupled with a Xevo G2-XS mass spectrometer (Waters Corp., Milford, MA, USA). An Acquity UPLC BEH C_18_ column (100 mm × 2.1 mm, 1.7 *μ*m) was employed. The temperature of column and autosampler were maintained at 35°C and 8°C, respectively. The mobile phase was consisted of 0.1% formic acid in acetonitrile as solvent A and 0.1% formic acid in water as solvent B. And, the following elution gradient was used: 0–2 min, 35–45% A; 2–10 min, 45–48% A; 10-11 min, 48–100% A; 11-12 min, 100-100% A; 12–12.01 min 100-35% A; and 12.01–15 min, 35-35% A. The flow rate was set to 0.40 mL/min, and the injection volume was 5 *μ*L.

The mass spectrometer was performed in the negative MS^E^ mode with a mass range from 50 to 1200 Da. The detection parameters of the ESI source were used as follows: capillary voltage, 2.5 kv; sample cone, 40 V; source offset, 80 V; source temperature, 100°C; flow rate of cone gas, 50 L/h; temperatures and flow rate of desolvation gas (N_2_), 400°C and 800 L/h; and collision energy, 2 eV in the low energy function and 10 to 30 eV in the high energy function. The software of MassLynx4.1 was used to control the instrument and acquire data.

### 2.5. UPLC-QqQ-MS/MS Conditions

The UPLC-QqQ-MS/MS analysis was carried out on an Acquity H-Class UPLC system (Waters Corp., Milford, MA, USA) coupled with a Xevo TQ-D mass spectrometer (Waters Corp., Milford, MA, USA). The UPLC conditions were similar to conditions of UPLC-Q/TOF-MS/MS. The mass spectrometer with ESI source was also used in the negative mode. Quantitation was carried out in the multiple reaction monitoring (MRM) mode. The detection parameters of the ESI source were used as follows: capillary voltage, 3.0 kv; cone voltage, 50 V; flow rate of cone gas (N_2_), 50 L/h; and temperatures and flow rate of desolvation gas (N_2_), 450°C and 900 L/h. The cone voltage and collision energy of twelve bile acids and IS were optimized by direct infusion into the MS system, respectively. The detailed parameters are listed in Table 2. The software of MassLynx4.1 was also used to control the instrument and acquire and analyze data.

### 2.6. Method Validation of the UPLC-QqQ-MS/MS

The linearity of the method was constructed by plotting the peak area ratio of the twelve compounds to IS versus their concentration. Each calibration curve was performed with six appropriate concentrations in duplicate. At the same time, the reference standard solution was gradually diluted and detected. The limits of quantitation (LOQs) were determined as the concentration whose S/N was 10, and limits of detection (LODs) were determined as the concentration whose S/N was 3.

The intra- and interday variations were chosen to evaluate the precision of the method. The mixed standard solutions were determined by six replicates within a day for the intraday variability test, while the mixed standard solutions were examined in consecutive three days for the interday variability test. Six copies of sample (20171211) were used to prepare the solution and investigate the repeatability of the method. And one of the solutions was also periodically analyzed at 0, 2, 4, 8, 12, and 24 h to evaluate the stability of the method.

To evaluate the accuracy of this method, a recovery test was performed. Three known amounts (low, middle, and high) of the twelve standards were added to the sample of no. 20171211. Then, the samples were extracted and analyzed using the aforementioned method, and triplicate experiments were performed at each level. Recovery of each analyte was calculated according to the following formula: recovery (%) = (found amount − original amount)/added amount × 100.

### 2.7. Quantification Analysis

The 20 batches of samples were collected from the main producing area of BA in China. Sample preparation and determination were the same as the aforementioned procedure. All the experiments were performed at least in triplicate with constant results.

### 2.8. Optimize the Fermentation Time of Bile Arisaema

The powder of *Arisaema amurense* Maxim and pig bile was mixed well in a ratio of 1 : 2. Then, the mixture was equally divided into ten portions and fermented in a constant temperature and humidity cabinet (Jing-Hong, Shanghai, China) at 37°C and 80% humidity. During the fermentative process, a mixture was randomly fetched on 0, 1, 3, 5, 7, 10, 15, 20, 25, and 30 days. And then, the mixture was steamed for 2 hours and dried at 40°C. Furthermore, the pig bile without Arisaema as the control group was fermented at the same time. Then, the components of twelve cholic acids in each fermentation points of BA and pig bile were determined.

## 3. Results and Discussion

### 3.1. Optimization of the Chromatographic and Spectrometric Conditions

The twelve analytes were firstly detected by UPLC-Q/TOF-MS/MS in both positive and negative ionization modes. It showed that the sensitivity and intensity of analyte signals obtained from the negative ion mode were higher than those from the positive ion mode. Thus, the ESI^−^ mode was selected for qualitative analysis of twelve compounds. To obtain the suitable fragment and product ions, the collision energy was optimized to 10–30 eV.

**Figure 2:**
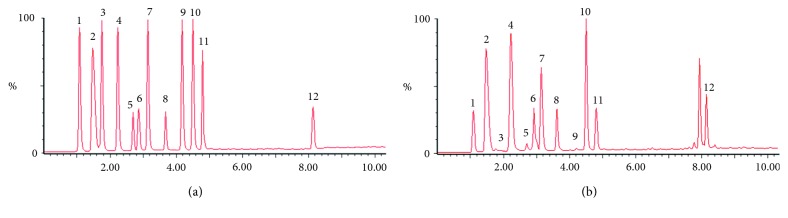
The typical UPLC-Q/TOF-MS/MS chromatogram of (a) mixed standards and (b) samples in Bile Arisaema (1, THCA; 2, THDCA; 3, TCA; 4, GHCA; 5, GCA; 6, GHDCA; 7, TCDCA; 8, HCA; 9, CA; 10, GCDCA; 11, HDCA; 12, CDCA).

To obtain satisfactory chromatographic separations, several UPLC analytical parameters were optimized. An Acquity UPLC BEH C_18_ column was selected, and the optimal mobile phase consisting of acetonitrile (0.1% formic acid) and water (0.1% formic acid) was finally employed. The gradient elution procedure was optimized, and it was also suggested that the separation was operated at the flow rate of 0.4 mL/min and the column temperature at 35°C. The typical chromatogram of standards and samples is shown in Figure 2.

**Figure 3:**
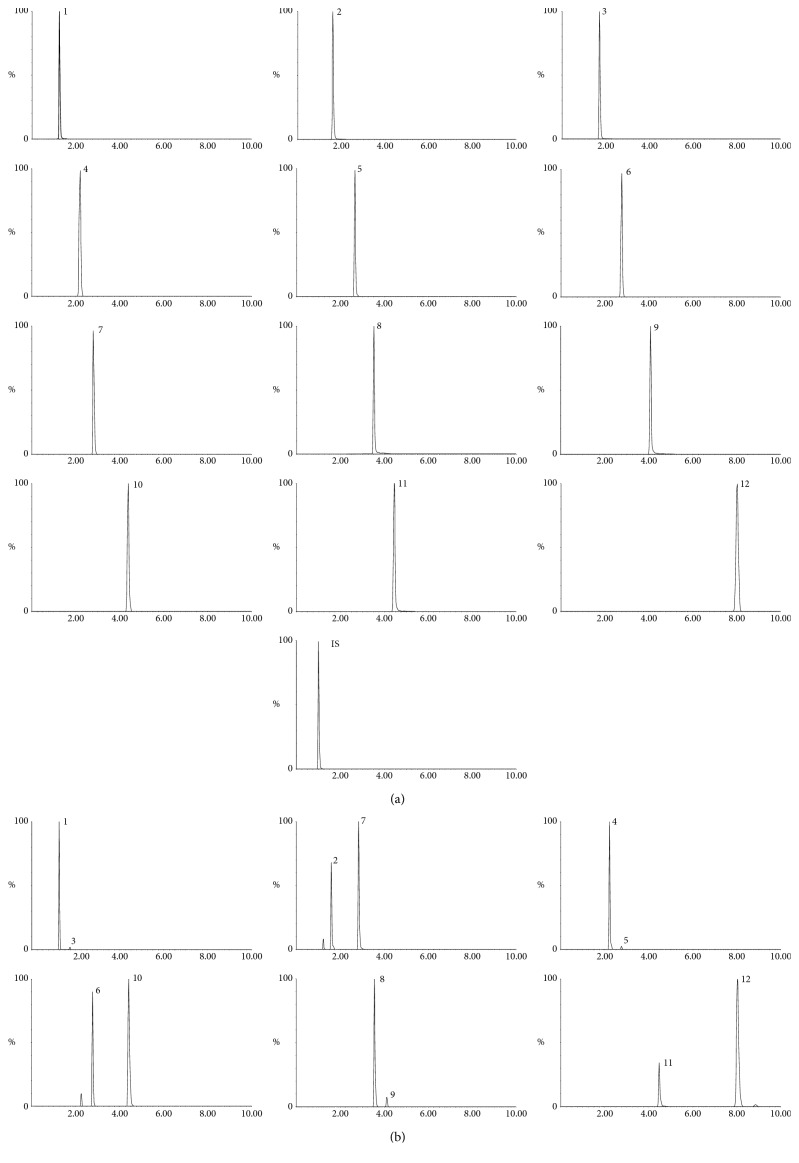
UPLC-QqQ-MS/MS multiple reaction mode (MRM) chromatograms of (a) mixed standards and (b) representative sample of BA (1, THCA; 2, THDCA; 3, TCA; 4, GHCA; 5, GCA; 6, GHDCA; 7, TCDCA; 8, HCA; 9, CA; 10, GCDCA; 11, HDCA; 12, CDCA) and IS, Ginsenoside Rh1.

As a result of UPLC-Q/TOF-MS/MS, the target compositions were quantitated by UPLC-QqQ-MS/MS in the negative ionization mode. The twelve analytes were detected by the direct full scan mass spectrometry method, and the deprotonated molecules [M-H]^−^ were selected as precursor ions. To obtain the maximum response of precursor and product ions, the parameters of fragment voltage and collision energy were further optimized. All the MRM transitions and parameters applied in the study are shown in Table 2. Under the above-optimized UPLC-QqQ-MS/MS conditions, all the twelve bile acids and IS could be separated satisfactorily within 10 min. The typical chromatograms are presented in Figure 3.

### 3.2. Qualitative Analysis of the Analytes by UPLC-Q/TOF-MS/MS

According to the type of structure, the twelve bile acids could be divided into free bile acids and the conjugated bile acids. In addition, it included two pairs of isomers (CA and HCA; HDCA and CDCA) in free bile acids and four pairs of isomers (GCA and GHCA; TCA and THCA; THDCA and TCDCA; GHDCA and GCDCA) in conjugate bile acids. In this study, the deprotonated molecule [M-H]^−^ was detected in the MS/MS spectra of all the analytes within 5.0 ppm (Tables 3 and 4). Besides, the free bile acids could produce high abundance of [M-H-nH_2_O]^−^ product ions, and the number of dehydration was the same as the hydroxyl number in the structure. The main and typical product ions include [M-H-CO_2_]^−^, [M-H-H_2_CO_2_]^−^, [M-H-H_2_CO_2_-H_2_O]^−^, and [M-H-H_2_O-C_5_H_8_O_2_]^−^. The typical side chain product ions [M-H-H_2_O-H_2_CO_2_]^−^ were significant to identify the structure of compounds. And, more remarkably, the product ion included losses of a 2H fragment, which was also a typical MS/MS feature in free bile acids.

**Figure 4:**
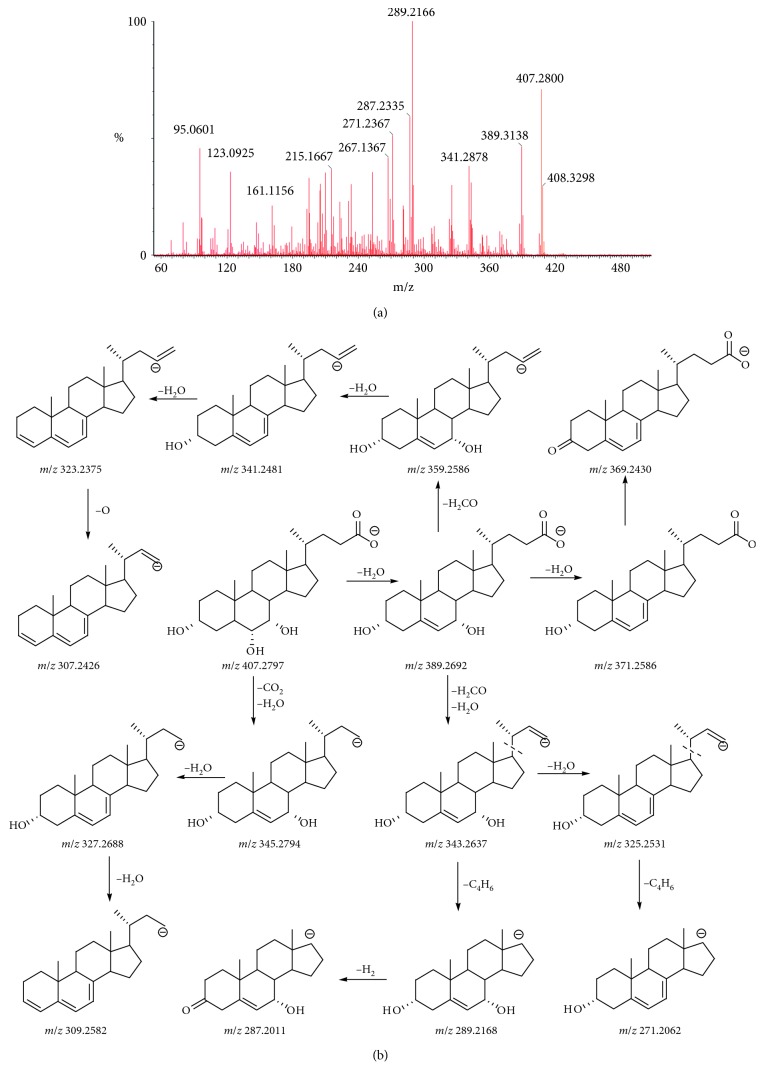
MS/MS spectra (a) and the proposed fragmentation pathway (b) of HCA.

The typical MS/MS spectra and fragmentation pathway of HCA are shown in Figure 4. The main and typical ions of this compound were the product ions of *m*/*z* 389.2697 [M-H-H_2_O]^−^, *m*/*z* 371.2594 [M-H-2H_2_O]^−^, *m*/*z* 353.2483 [M-H-3H_2_O]^−^, *m*/*z* 363.5540 [M-H-CO_2_]^−^, *m*/*z* 361.5381 [M-H-H_2_CO_2_]^−^, and *m*/*z* 343.2642 [M-H-H_2_CO_2_-H_2_O]^−^, which corresponded to the losses of a series of H_2_O and one molecule of CO_2_ (44 Da), H_2_CO_2_ (46 Da), H_2_CO_2_-H_2_O (64 Da), and H_2_O-C_5_H_8_O_2_ (118 Da). The product ion of *m*/*z* 389.2697 [M-H-H_2_O]^−^ would continually dehydrate to product the ion of *m*/*z* 345.2794 [M-H-CO_2_-H_2_O]^−^ and *m*/*z* 309.2582 [M-H-CO_2_ -2H_2_O]^−^. The product ion of *m*/*z* 361.5381 [M-H-H_2_CO_2_]^−^ would continually dehydrate to product the ion of *m*/*z* 343.2642 [M-H-H_2_CO_2_-H_2_O]^−^ and *m*/*z* 325.2537 [M-H-H_2_CO_2_-2H_2_O]^−^ also. The [M-H-H_2_O-H_2_CO_2_]^−^ will lose the side chain and product the ions *m*/*z* 289.2166 [M-H-H_2_O-C_5_H_8_O_2_]^−^. These product ions also had a lot of losses of 2H fragment ions like *m*/*z* 341.2481, *m*/*z* 323.2375, and *m*/*z* 287.2011. In addition, there were differences of products ions and abundance ratio for isomers of the other free bile acids. The detailed mass data of the five free bile acids are listed in Table 3.

**Figure 5:**
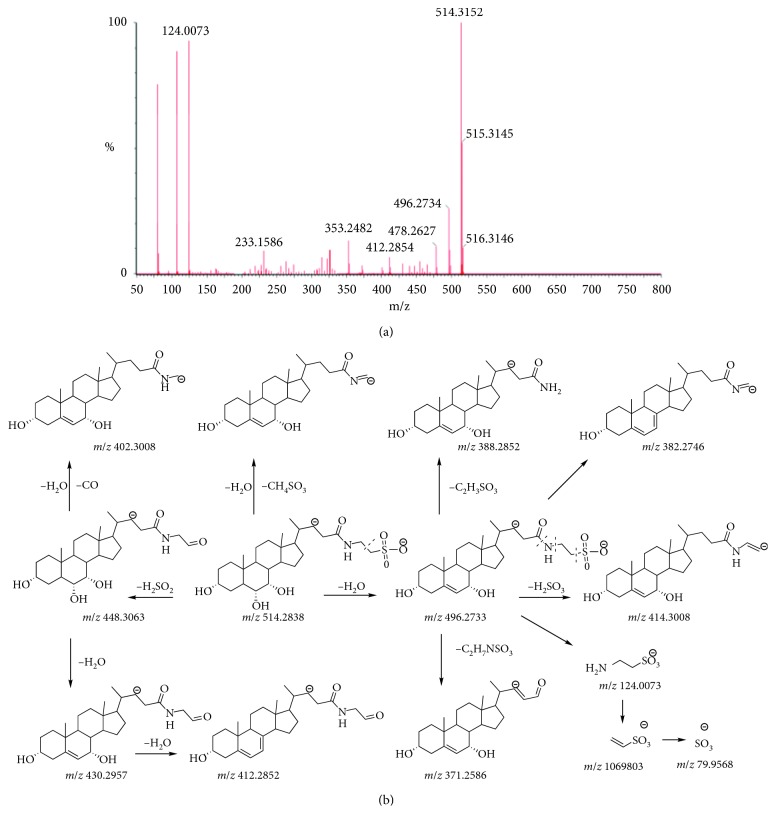
MS/MS spectra (a) and the proposed fragmentation pathway (b) of THCA.

**Figure 6:**
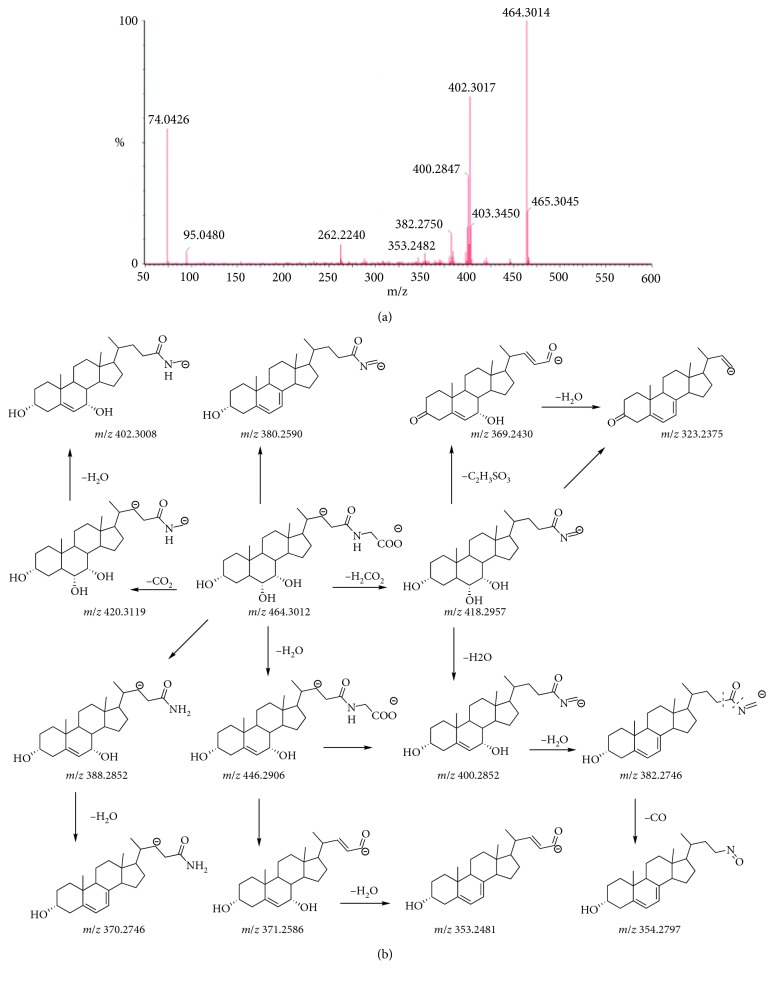
MS/MS spectra (a) and the proposed fragmentation pathway (b) of GHCA.

The conjugate bile acids could fall into taurine and glycine type according to the kind of binding amino acid also. Except the deprotonated molecule [M-H]^−^ detected in the MS/MS spectra, the bile acids of the taurine type could produce high abundance fragment ions of [SO_3_]^−^, [NH_2_-CH_2_-CH_2_-SO_3_]^−^, and [CH_2_=CH_2_-SO_3_]^−^. Moreover, it could lose the fragment of 66 Da, 82 Da, 94 Da, 96 Da, 108 Da, and 125 Da and product the typical ions of [M-H-H_2_SO_2_]^−^, [M-H-H_2_SO_3_]^−^, [M-H-CH_2_SO_3_]^−^, [M-H-CH_4_SO_3_]^−^, [M-H-C_2_H_4_SO_3_]^−^, and [M-H-C_2_H_7_NSO_3_]^−^. The bile acids of the glycine type could produce high abundance fragment ions of [NH_2_-CH_2_-COO]^−^. It could lose the fragment of 44 Da, 46 Da, 58 Da, and 75 Da and product the typical ions of [M-H-CO_2_]^−^, [M-H-H_2_CO_2_]^−^, [M-H-C_2_H_2_O_2_]^−^, and [M-NH_2_CH_2_COO]^−^. The MS/MS spectra and fragmentation pathway of THCA and GHCA are shown in Figures 5 and 6. In addition, there were differences of products ions and abundance ratio for isomers of the other conjugate bile acids. The detailed mass data of the eight conjugate bile acids are listed in Table 4.

### 3.3. Validation of the Quantitative Methods of UPLC-QqQ-MS/MS

Quantitative method was validated by evaluating the linearity, precision, limit of detection (LOD), limit of quantification (LOQ), repeatability, and stability. All results are listed in Table 5. The calibration curves of twelve compounds exhibited relatively wide concentration ranges with correlation coefficients higher than 0.9989. The intra- and interday precisions of the components exhibited RSD of less than 2.99% and 4.25%, respectively. For all ingredients, the LODs ranged from 0.56 to 28.17 ng/mL and the LOQs from 0.75 to 84.5 ng/mL. The repeatability and stability of the components presented as RSD were in the range from 3.27 to 4.12 and from 1.71 to 4.25. In addition, the average recoveries of these compounds were in the range of 95.36–102.15% (Table 6) indicating that the proposed method had good reliability and accuracy. Compared with the previous analysis method, it had higher sensitivity and shorter analysis time. It was the first time to detect twelve bile acids in BA by the same system [9–11].

### 3.4. Quantification of the Twelve Components in Different Regions of Bile Arisaema

The validated UPLC-QqQ-MS/MS method was applied to simultaneously quantify the twelve compounds in twenty batches of BA samples collected from different regions in China. Table 1 gives a summary of the content of twelve analytes from these samples. The results showed that the target compounds of twelve bile acids were varied obviously in samples from various origins. It indicated the serious quality problems of BA in the market. In fact, it had been reported in many other studies [10, 11]. Except for the reasons of artificial counterfeit, one of the main reasons was the processing method of BA which included the origin of raw material and fermentation time having huge differences in different areas. Thus, it was very important to establish a uniform and standard processing method which could ensure the clinical curative effect of BA. It was also needed to optimize the processing technology and explore the processing mechanism of BA as a premise.

### 3.5. Optimize the Fermentation Time and Explore the Processing Mechanism

**Figure 7:**
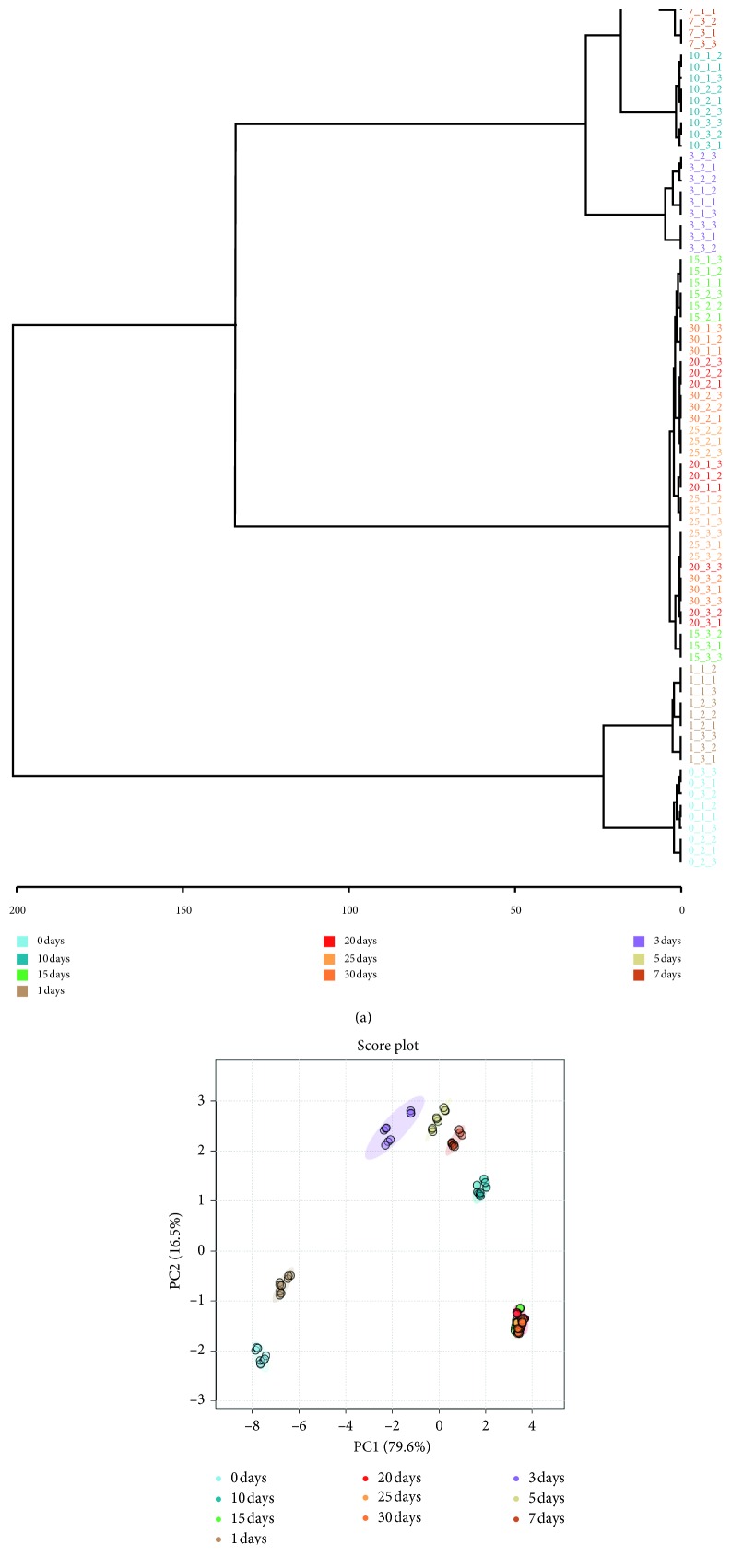
(a) HCA of different fermentation times of Bile Arisaema; (b) PCA of different fermentation times of Bile Arisaema.

In order to optimize the fermentation technology of BA, the same sample of BA which had different fermentation times was determined. HCA (hierarchical clustering analysis) and PCA (principal components analysis) were performed on the basis of the content of twelve bile acids compounds from UPLC-QqQ-MS/MS profiles by employing MetaboAnalyst 4.0 software (http://www.metaboanalyst.ca). The dendrogram of HCA is shown in Figure 7(a) where it could be seen that the 90 samples were spread over 10 different fermentation times and grouped into two main clusters. The samples of 0 day and 1 day were clustered to one group, indicating that the BA did not begin to ferment. Other samples were further clustered into two corresponding subgroups, respectively. The samples of 3 days, 5 days, 7 days, and 10 days were clearly clustered to one group, and the samples of 15 days, 20 days, 25 days, and 30 days were clustered to another group. It indicated that the BA would show significant changes in 15 days of fermentation. The score plot of PCA is shown in Figure 7(b), from which the degree of fermentation could be revealed more clearly. The first two principal components (PC1 and PC2) with >96% of the whole variance were extracted for analysis. Among them, PC1 accounted for 79.60% of total variance, whereas PC2 explained 16.50 of total variance. In the score plot, each sample was represented as a marker and each color was represented as a fermentation times. It could be seen that the samples of 0 days, 1 days, 3 days, 5 days, 7 days, and 10 days were clearly clustered to one group, respectively. From 15 days to 30 days, all the samples were gathered together which demonstrated that BA could complete fermentation in 15 days. The detailed content of twelve bile acids compounds is also listed in Table 7. From this table, it could be seen that there was no significant change in the content of twelve bile acids compounds after fermentation for 15 days. This result was also consistent with the above multivariate statistical analysis. So, it could be speculated that the fermentation time of BA was 15 days.

In Table 7, it was also shown that the conjugate bile acids were the major chemical constituents and the free bile acids were rarely detected in unfermentable BA. After fermentation, the content of free bile acids significantly increased and the conjugate bile acids decreased significantly in BA. This was in accordance with the above results in Table 1 which included the content of 12 bile acids in BA samples collected from main regions in China. Furthermore, in order to ascertain the mechanism of above phenomenon, the content of bile acids in the pig bile without Arisaematis during fermentation was determined. The detailed results are also listed in Table 7. These results indicated that the content of free bile acids in the pig bile without Arisaematis has not significantly increased as BA during fermentation. In fact, free bile acids had better efficacy of clearing heat and reducing phlegm than the conjugate bile acids which could enhance the clinical efficacy of BA. Thus, the aim of mixed fermentation by the Arisaematis and pig bile might promote the conjugated bile acids to transform into free bile acids in pig bile. It could be one of possible processing mechanisms of Bile Arisaema.

## 4. Conclusions

In this study, the proposed fragmentation behaviors of the bile acids were illuminated. It could provide a reference for screening bile acids in Bile Arisaema due to similarity in their skeleton and fragment groups. A simple, sensitive, and feasible UPLC-QqQ-MS/MS method was developed and validated for the simultaneous determination of twelve bile acids in Bile Arisaema. The developed method offered the advantages of simple sample preparation and high sensitivity. It was successfully applied to simultaneously quantify the twelve bioactive components in twenty batches of Bile Arisaema samples collected from different regions of China. In addition, comparative analysis of twelve bioactive components in different fermentation times which confirmed the time of 15 days was suitable for BA. Furthermore, one of the possible processing mechanisms of BA was promoting the conjugated bile acids to transform into free bile acids.

## Figures and Tables

**Table 1:** Quantitative analytical results of Bile Arisaema in commercial (*μ*g/g, *n* = 3).

Number	Origins	HDCA	CA	CDCA	HCA	GCDCA	GCA	GHDCA	TCDCA	THDCA	TCA	GHCA	THCA
20171201	Sichuan C&Y Traditional Chinese medicine Co., Ltd. (LOT.160109)	1950.70	27.61	2833.93	870.70	62.74	6.12	47.23	13.98	9.12	5.85	22.34	11.25
20171202	Sichuan C&Y Traditional Chinese Medicine Co., Ltd. (LOT.160101)	2121.04	29.19	3002.56	935.81	26.42	8.15	21.72	15.32	11.23	6.55	31.18	24.29
20171203	Sichuan C&Y Traditional Chinese Medicine Co., Ltd. (LOT.160601)	2101.06	33.45	3136.33	969.50	23.10	4.36	17.01	12.36	10.37	4.96	24.03	15.49
20171204	Sichuan Fuzheng Pharmaceutical Co., Ltd. (LOT.160509)	104.19	4.41	212.17	68.38	2.14	0.19	3.73	5.45	0.57	2.54	2.06	1.29
20171205	Sichuan Fuzheng Pharmaceutical Co., Ltd. (LOT.161206)	78.62	2.06	169.29	61.11	5.12	1.26	5.64	4.88	3.59	0.59	1.21	3.44
20171206	Sichuan Fuzheng Pharmaceutical Co., Ltd. (LOT.170108)	122.40	5.24	235.92	76.29	0.16	2.16	3.21	0.57	1.26	0.22	1.09	3.64
20171207	Neijiang Lianghui Pharmaceutical Co., Ltd. (LOT.170302)	ND	ND	ND	ND	ND	ND	2.68	ND	ND	ND	ND	ND
20171208	Beijing Tongrentang (Bozhou) Slice Co., Ltd. (LOT. 601002551)	326.73	2.75	155.33	178.06	2.58	1.26	0.59	4.22	3.87	0.34	4.25	3.51
20171209	Anhui Jiayu Traditional Chinese Medicine Co., Ltd. (LOT. 170206)	28.62	2.86	30.45	35.89	79.49	3.26	61.49	71.60	4.89	0.79	3.16	2.53
20171210	Beijing Huamiao Pharmaceutical Co., Ltd. (LOT. SBB2661)	834.94	6.53	1928.04	637.88	14.86	6.39	63.18	13.95	36.25	51.28	30.24	28.56
20171211	Beijing Huamiao Pharmaceutical Co., Ltd. (LOT. SB6191)	313.17	15.31	1048.05	295.01	5032.17	154.92	3436.47	750.92	258.18	197.53	146.55	164.23
20171212	Anguo Juyaotang Pharmaceutical Co., Ltd. (LOT. 1701002)	354.21	7700.96	1013.32	8327.74	43.26	328.29	132.1	562.37	78.16	1121.39	129.5	821.28
20171213	Sichuan Baisheng Pharmaceutical Co., Ltd. (LOT.170902)	4216.25	47.62	6386.69	2287.49	16.35	156.87	253.19	113.44	102.51	15.26	27.34	11.25
20171214	Sichuan Baisheng Pharmaceutical Co., Ltd. (LOT.171204)	839.32	21.16	1361.54	511.98	700.56	46.57	662.99	176.89	58.12	39.05	24.35	19.72
20171215	Sichuan Qianfang Traditional Chinese Medicine Co., Ltd. (LOT. 20170601)	4165.87	32.53	4444.88	1724.25	172.47	126.34	367.21	184.37	85.25	88.52	118.23	59.69
20171216	Sichuan Jianqu Pharmaceutical Co., Ltd. (LOT.170920)	2274.11	24.35	2133.02	1117.43	102.39	52.16	18.82	110.29	49.38	89.15	52.67	47.26
20171217	Yibin Traditional Chinese Medicine Co., Ltd. (LOT. 170501)	ND	5.45	ND	31.98	8.20	ND	9.05	ND	ND	ND	ND	ND
20171218	Sichuan Hongsheng Pharmaceutical Co., Ltd. (LOT.170903)	494.08	19.66	282.36	252.93	5.32	2.23	8.69	7.56	9.16	4.61	6.56	5.31
20171219	Sichuan Guanghan Traditional Chinese Medicine Co., Ltd. (LOT. 170411)	412.64	3607.23	918.88	3882.89	146.58	23.59	253.46	108.16	62.04	136.77	153.4	103.72
20171220	Beijing Qiancao Traditional Chinese Medicine Co., Ltd. (LOT. 170806)	660.79	536.16	1552.12	3818.08	100.23	55.61	165.24	253.97	176.24	248.56	236.12	118.55

ND: not detected.

**Table 2:** The optimized MRM parameters and transitions for each analyst in UPLC-QqQ-MS/MS.

Analyte	*t*_R_ (min)	[M-H]^−^(*m*/*z*)	MRM transitions (precursor ⟶ product)	Cone voltage (V)	Collision energy (eV)
(1) THCA	1.25	514.39	514.39 ⟶ 80.14	100.0	66.0
(2) THDCA	1.60	498.39	498.39 ⟶ 80.14	100.0	65.0
(3) TCA	1.74	514.39	514.39 ⟶ 80.14	100.0	66.0
(4) GHCA	2.20	464.40	464.40 ⟶ 74.10	76.0	38.0
(5) GCA	2.74	464.40	464.40 ⟶ 74.10	76.0	38.0
(6) GHDCA	2.77	448.41	448.41 ⟶ 74.10	74.0	36.0
(7) TCDCA	2.83	498.39	498.39 ⟶ 80.14	100.0	65.0
(8) HCA	3.55	407.31	407.31 ⟶ 343.42	78.0	30.0
(9) CA	4.12	407.31	407.31 ⟶ 343.42	78.0	30.0
(10) GCDCA	4.41	448.41	448.41 ⟶ 74.10	74.0	36.0
(11) HDCA	4.47	391.35	391.35 ⟶ 345.49	80.0	34.0
(12) CDCA	8.04	391.35	391.35 ⟶ 345.49	80.0	34.0
(13) Ginsenoside Rh1	1.05	637.43	637.43 ⟶ 475.26	100.0	40.0

**Table 3:** Mass data of the four free bile acids from Bile Arisaema by UPLC-Q/TOF-MS/MS.

Compound	Formula	Predicted mass (*m*/*z*)	Measured mass (*m*/*z*)	Error (ppm)	MS^2^ (*m*/*z*)		Side chain eliminated fragments
					Lose H_2_O fragments	Lose CO, CO_2_, and H_2_CO_2_ fragments	
CA	C_24_H_40_O_5_	407.2797	407.2800 [M-H]^−^	+1.2	[407]: 389.2697, 371.2588, 353.2483	369.2433, 345.2800, 343.2635, 341.243, 325.2537, 323.2379, 309.2588	*289*.*2166*, 287.2010, 271.2064, 253.1960
HCA	C_24_H_40_O_5_	407.2797	407.2800 [M-H]^−^	+1.2	[407]: 389.2697, 371.2588, 353.2483	345.2800, 343.2635, 341.2500, 327.2693, 323.2379, 309.2588	*289*.*2166*, 271.2064, 253.1960
CDCA	C_24_H_40_O_4_	391.2848	391.2853 [M-H]^−^	+1.3	[*391*]: 373.2744, 355.2637	343.2638, 329.2839, 327.2685, 325.2533, 299.2366	273.2219, 271.2065, 255.2115
HDCA	C_24_H_40_O_4_	391.2848	391.2853 [M-H]^−^	+1.3	[*391*]: 373.2743, 355.2637	343.2638, 329,2839, 327.2685, 325.2533, 299.2366	287.2379, 273.2219, 171.2065, 255.2115

^∗^Base peaks are represented in italics.

**Table 4:** Mass data of the eight conjugate bile acids from Bile Arisaema by UPLC-Q/TOF-MS/MS.

Compounds	Formula	Predicted mass (*m*/*z*)	Measured mass (*m*/*z*)	Error (ppm)	MS^2^ (*m*/*z*)		
					Lose H_2_O fragments	Side chain eliminated fragments	Taurine/glycine fragments
TCA	C_26_H_45_NO_7_S	514.2838	514.2841 [M-H]^−^	+0.6	[*514*]: 496.2737, 478.2631	448.3066, 430.2963, 412.2855, 402.3011, 400.2856, 382.275, 371.2590	79.9573, 106.9806, 124.0073
THCA	C_26_H_45_NO_7_S	514.2838	514.2841 [M-H]^−^	+0.6	[514]: 496.2737, 478.2631	448.3066, 430.2963, 412.2855, 402.3011, 382.2751, 371.2590	79.9573, 106.9806, *124*.*0073*
TCDCA	C_26_H_45_NO_6_S	498.2889	498.2891 [M-H]^−^	+0.6	[*498*]: 480.2788, 462.2680	432.3118, 414.3010, 386.3061, 373.2748, 368.259, 355.2640	79.9573, 106.9808, 124.0073
THDCA	C_26_H_45_NO_6_S	498.2889	498.2891 [M-H]^−^	+0.6	[*498*]: 480.2788, 462.2680	432.3118, 414.3010, 386.3061, 373.2748, 368.259, 355.2640	79.9573, 106.9809, 124.0072
GCA	C_26_H_43_NO_6_	464.3012	464.3016 [M-H]^−^	+0.9	[464]: 446.2929	418.2961, 402.3012, 371.2593, 369.2430, 354.2612, 323.2377	*74*.*0247*
GHCA	C_26_H_43_NO_6_	464.3012	464.3016 [M-H]^−^	+0.9	[*464*]: 446.2929	418.2961, 402.3012, 371.2593, 369.2430, 354.2612, 323.2377	74.0245
GCDCA	C_26_H_43_NO_5_	448.3063	448.3068 [M-H]^−^	+1.1	[448]: 430.2962	404.3168, 402.3007, 386.3065, 368.2958	*74*.*0243*
GHDCA	C_26_H_43_NO_5_	448.3063	448.3068 [M-H]^−^	+1.1	[448]: 430.2962	404.3168, 386.3065, 368.2958	*74*.*0243*

^∗^Base peaks are presented in italics.

**Table 5:** Calibration curves, linearity ranges, limit of detection (LOD), limit of quantification (LOQ), precision, repeatability, and stability of the twelve analytes.

Analytes	Calibration curves	*R* ^2^	Linear range (*μ*g/mL)	Precision (RSD, %)		LOQ (ng/mL)	LOD (ng/mL)	Repeatability (RSD, %, *n* = 6)	Stability (RSD, %, *n* = 6)
				Intraday	Interday				
(1) THCA	*Y* = 6928.5*X* + 233.51	0.9998	0.22–8.90	1.78	3.13	2.22	0.56	4.05	2.64
(2) THDCA	*Y* = 7196.9*X* + 2433.4	0.9996	0.68–27.20	0.62	2.52	2.72	0.68	4.12	2.72
(3) TCA	*Y* = 2615.2*X* + 70.046	0.9997	0.27–10.60	2.33	3.40	5.30	1.33	3.29	4.25
(4) GHCA	*Y* = 3991.9*X* + 233.63	0.9989	0.14–2.80	2.86	3.11	5.60	1.40	3.57	4.19
(5) GCA	*Y* = 57819*X* + 4483.4	0.9982	0.15–3.00	0.82	1.42	0.75	0.19	4.04	2.35
(6) GHDCA	*Y* = 9643.4*X* + 652.74	0.9990	0.16–3.15	0.79	1.96	3.15	0.79	3.54	1.71
(7) TCDCA	*Y* = 2210.5*X* + 223.69	0.9997	0.55–21.90	1.93	3.34	6.84	2.19	3.43	2.99
(8) HCA	*Y* = 10575*X* + 110.15	0.9994	0.20–7.80	2.18	4.25	2.17	0.78	3.54	3.90
(9) CA	*Y* = 2408.1*X* + 4.2004	0.9991	0.16–3.25	2.99	4.01	8.13	1.63	3.27	3.97
(10) GCDCA	*Y* = 7134.9*X* + 1131.2	0.9987	0.49–9.85	1.13	1.53	4.93	1.23	4.11	2.19
(11) HDCA	*Y* = 25067*X* + 2257.9	0.9989	0.22–4.40	0.85	2.43	8.80	2.20	3.65	3.82
(12) CDCA	*Y* = 5537.9*X* + 920.54	0.9985	0.42–8.45	1.49	1.98	84.50	28.17	3.81	1.78

**Table 6:** Recoveries of the twelve compounds (*n* = 3).

Analytes	Samples (g)	Origin (*μ*g)	Spiked (*μ*g)	Found (*μ*g)	Mean recovery (%) (RSD, %)
(1) THCA	1.0	52.13	42.72	94.41	99.23 (1.11)
			53.40	104.52	98.27 (1.79)
			64.08	115.56	97.10 (2.96)

(2) THDCA	1.0	103.35	82.69	185.95	99.90 (1.95)
			103.36	205.63	98.93 (2.43)
			124.03	227.24	95.36 (2.10)

(3) TCA	1.0	70.48	56.82	126.41	98.73 (2.30)
			71.02	143.35	97.87 (1.46)
			85.22	154.87	99.07 (2.13)

(4) GHCA	1.0	28.20	22.40	50.42	99.13 (0.96)
			28.00	56.05	96.30 (1.97)
			33.60	61.37	98.63 (2.49)

(5) GCA	1.0	28.35	23.04	51.42	100.63 (0.66)
			28.80	56.53	97.90 (1.77)
			34.56	62.50	96.17 (0.96)

(6) GHDCA	1.0	264.26	211.68	476.52	100.33 (0.90)
			264.60	523.14	97.87 (1.76)
			317.52	580.71	98.67 (2.30)

(7) TCDCA	1.0	270.41	219.00	488.12	96.43 (1.99)
			273.75	544.43	102.15 (0.65)
			328.50	593.45	98.30 (1.99)

(8) HCA	1.0	31.11	24.96	55.63	98.43 (1.12)
			31.20	62.12	97.10 (1.68)
			37.44	68.41	99.73 (2.21)

(9) CA	1.0	28.71	22.88	51.34	97.10 (1.36)
			28.60	57.18	98.73 (1.57)
			34.32	62.56	100.97 (1.53)

(10) GCDCA	1.0	651.34	520.08	1161.43	98.13 (1.48)
			650.10	1301.54	100.15 (1.45)
			780.12	1434.82	101.40 (1.08)

(11) HDCA	1.0	21.62	16.72	38.43	96.33 (0.85)
			21.12	42.71	98.73 (1.42)
			24.64	46.08	99.17 (0.44)

(12) CDCA	1.0	127.84	101.40	228.02	99.17 (0.78)
			126.75	253.71	97.60 (1.06)
			152.10	279.73	101.07 (1.50)

**Table 7:** Compare of the content of 12 bile acids between Bile Arisaema and pig bile in different fermentation times (*μ*g/g, *n* = 9).

Samples	HDCA	CA	CDCA	HCA	GCDCA	GCA	GHDCA	TCDCA	THDCA	TCA	GHCA	THCA
0 days	ND (ND)	ND (ND)	0.82 (1.35)	0.47 (0.58)	253.30 (297.43)	10.90 (12.36)	97.25 (123.46)	97.97 (134.19)	36.50 (45.36)	24.26 (34.26)	10.11 (15.26)	20.19 (29.38)
1 day	ND (ND)	ND (ND)	1.23 (1.42)	0.61 (0.69)	251.87 (295.67)	10.76 (12.22)	97.26 (123.23)	97.88 (133.99)	36.45 (45.13)	24.21 (34.27)	10.09 (15.29)	20.11 (29.41)
3 days	10.25 (ND)	ND (ND)	2.46 (1.54)	0.96 (0.72)	230.34 (289.37)	9.55 (12.17)	96.87 (120.39)	96.26 (132.45)	35.68 (45.09)	23.89 (33.75)	9.86 (15.11)	20.03 (27.99)
5 days	50.21 (5.21)	ND (ND)	40.55 (2.57)	4.28 (0.93)	216.42 (285.63)	8.86 (12.06)	87.25 (118.54)	90.03 (130.58)	30.42 (44.18)	20.15 (33.45)	9.26 (15.03)	18.85 (26.45)
7 days	80.54 (11.49)	1.24 (ND)	88.75 (3.47)	8.21 (1.23)	197.57 (277.54)	8.13 (11.96)	77.52 (114.86)	77.25 (125.29)	21.73 (43.09)	18.52 (32.98)	8.55 (14.56)	15.56 (25.13)
10 days	110.37 (20.18)	2.37 (ND)	187.89 (8.24)	20.34 (1.46)	186.25 (265.12)	7.46 (11.68)	68.39 (110.43)	54.29 (121.36)	11.51 (42.58)	14.75 (31.52)	7.23 (13.87)	10.23 (24.02)
15 days	142.48 (31.11)	3.65 (ND)	376.88 (12.31)	84.00 (1.57)	153.39 (254.31)	6.78 (11.27)	55.84 (105.16)	45.83 (118.33)	5.40 (41.16)	12.54 (30.37)	6.27 (12.45)	6.70 (23.45)
20 days	146.21 (42.15)	4.17 (ND)	388.03 (20.15)	88.14 (1.89)	151.76 (246.77)	6.62 (10.79)	54.46 (100.29)	45.26 (112.20)	5.26 (38.73)	12.26 (28.32)	6.12 (11.01)	6.43 (21.36)
25 days	147.06 (53.32)	4.37 (ND)	391.16 (30.49)	90.91 (2.16)	149.11 (220.10)	6.43 (9.42)	53.92 (95.01)	45.09 (103.15)	5.12 (35.22)	12.14 (27.16)	6.03 (10.28)	6.29 (18.19)
30 days	149.35 (60.43)	4.49 (ND)	399.24 (50.49)	91.16 (3.26)	147.21 (201.23)	6.24 (8.79)	53.02 (90.12)	44.88 (95.37)	5.03 (30.11)	11.94 (25.46)	5.98 (8.99)	6.11 (16.33)

ND, not detected; (), content of bile acids in the pig bile.

## Data Availability

The data used to support the findings of this study are included within the article.

## Abbreviations

BA: Bile Arisaema
UPLC: Ultrahigh-performance liquid chromatography
Q/TOF-MS: Quadrupole time-of-flight tandem mass spectrometry
QqQ-MS: Triple quadrupole mass spectrometry
MRM: Multiple reaction monitoring
HDCA: Hyodeoxycholic acid
CA: Cholic acid
CDCA: Chenodeoxycholic acid
HCA: Hyocholic acid
GCDCA: Glycochenodeoxycholic acid
GCA: Glycocholic acid
GHDCA: Glycohyodeoxycholic acid
TCDCA: Taurochenodeoxycholic acid
THDCA: Taurohyodeoxycholic acid
TCA: Taurocholic acid
GHCA: Glycohyocholic acid
THCA: Taurohyocholic acid
HCA: Hierarchical clustering analysis
PCA: Principal components analysis.
